# Hemoptysis as the Initial Presentation of a Ruptured Aortic Aneurysm: A Case Report and Literature Review

**DOI:** 10.7759/cureus.68898

**Published:** 2024-09-07

**Authors:** Juan Manuel Millan-Alanis, Martha Cecilia Jimenez-Leos, Alma Nelly Jaramillo-Amador, Magda Arredondo-Flores, Homero Náñez-Terreros

**Affiliations:** 1 Internal Medicine, Hospital Universitario Dr. Jose Eleuterio Gonzalez, Universidad Autonoma De Nuevo Leon, Monterrey, MEX; 2 Pulmonary and Critical Care Medicine, Hospital Universitario Dr. Jose Eleuterio Gonzalez, Universidad Autonoma De Nuevo Leon, Monterrey, MEX

**Keywords:** cardiovascular disease (cvd), clinical case report, descending aortic aneurysm, emergency medicine, life-threatening hemoptysis

## Abstract

Hemoptysis has a broad differential diagnosis, with common causes including bronchiectasis, bronchitis, pulmonary tuberculosis, and lung neoplasms. While often benign, it can sometimes indicate more severe, life-threatening conditions. Herein we report the case of an 86-year-old woman who presented to the emergency department with a 30-day history of hemoptysis ultimately leading to hemodynamic instability. She was initially admitted to the emergency department for resuscitation and diagnostic workup. During the hospitalization, we identified a large, ruptured aneurysm of the descending and diaphragmatic aorta contained by a hematoma communicating the tracheobronchial tree. This case highlights the importance of considering a broad differential diagnosis in patients presenting with hemoptysis as it can signal severe underlying conditions such as a ruptured aortic aneurysm. Early recognition and appropriate management of these cases are crucial to improving patient outcomes.

## Introduction

Hemoptysis is defined as the expectoration of blood originating from the lower respiratory tract. Its differential diagnosis is extensive, with the most common causes being bronchiectasis, bronchitis, pulmonary tuberculosis, and lung neoplasms. Although it is often non-life-threatening, it can sometimes be the initial presentation of more severe conditions (e.g., pulmonary embolism, severe pneumonia, diffuse alveolar hemorrhage, ruptured aortic aneurysm, etc.) [1-3].

Herein, we report the case of an 86-year-old woman diagnosed with a ruptured aortic aneurysm, who presented to the emergency department with a 30-day history of hemoptysis. This case represents a highly atypical presentation of a ruptured aortic aneurysm, making its reporting crucial for clinicians to consider this differential diagnosis in patients with hemoptysis of unknown origin.

## Case presentation

An 86-year-old female presented to the emergency department accompanied by her family, reporting a 30-day history of intermittent hemoptysis that had escalated on the day of admission. Her past medical history included a diagnosis of osteoarthritis, a laparoscopic cholecystectomy, and peptic ulcer disease treated with a therapeutic endoscopy. Due to severe frailty and functional impairment, she had been bedridden for the year prior to admission. She denied use of antiplatelet or anticoagulant agents.

Her symptoms began 30 days prior to admission with intermittent hemoptysis during the day. Family members described her symptoms as multiple episodes of coughing accompanied by small amounts of fresh, "reddish" blood. She denied the presence of chest pain, dyspnea, dizziness, fatigue, weight loss, night sweats, or bleeding from other sites. Two hours before arriving at the emergency room, she experienced a major episode of hemoptysis, along with approximately 10 other additional episodes as reported by her family. Upon admission, we found the following vital signs: blood pressure of 50/30 mmHg, heart rate of 134 beats per minute, respiratory rate of 22 breaths per minute, and oxygen saturation of 86% at room air.

Physical examination was unremarkable except for the previously mentioned findings. She was initially resuscitated with crystalloid solutions and blood products, raising her blood pressure to 100/60 mmHg. A complete blood count, comprehensive serum biochemistry, and venous blood gases were collected revealing hemoglobin of 9.91 g/dL and blood lactate of 4.3 mmol/L. During the next hour of her stay, she experienced another episode of hemoptysis (approximately 300 mL) accompanied by hemodynamic instability requiring two additional blood transfusions. No further episodes were documented.

**Figure 1:**
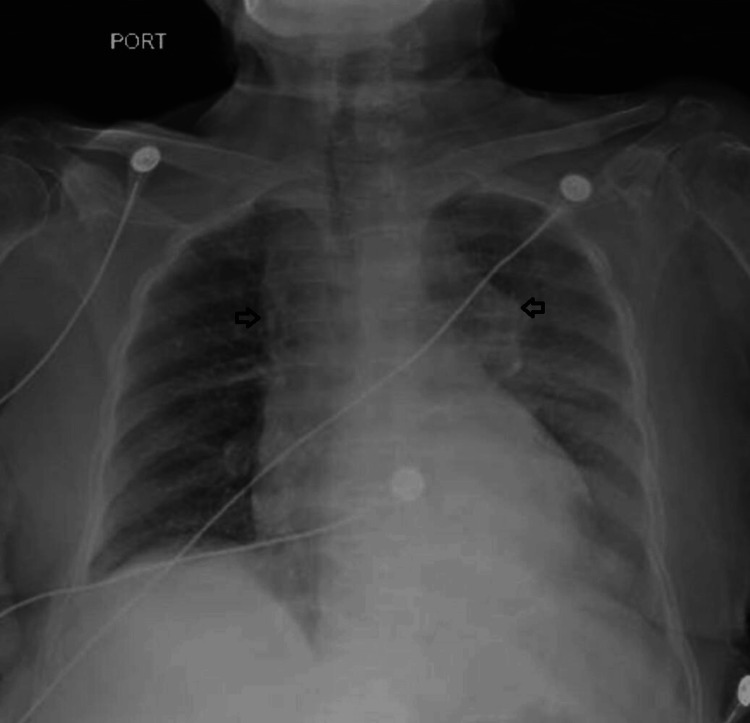
Initial anteroposterior chest X-ray displaying mediastinal widening (black arrows)

In the diagnostic assessment, an initial chest X-ray revealed mediastinal widening, an irregular radiopacity at the aortic arch level, and a diffuse, slight radiopacity in the left hemithorax with horizontalization of the left main bronchus (Figure 1).

**Figure 2:**
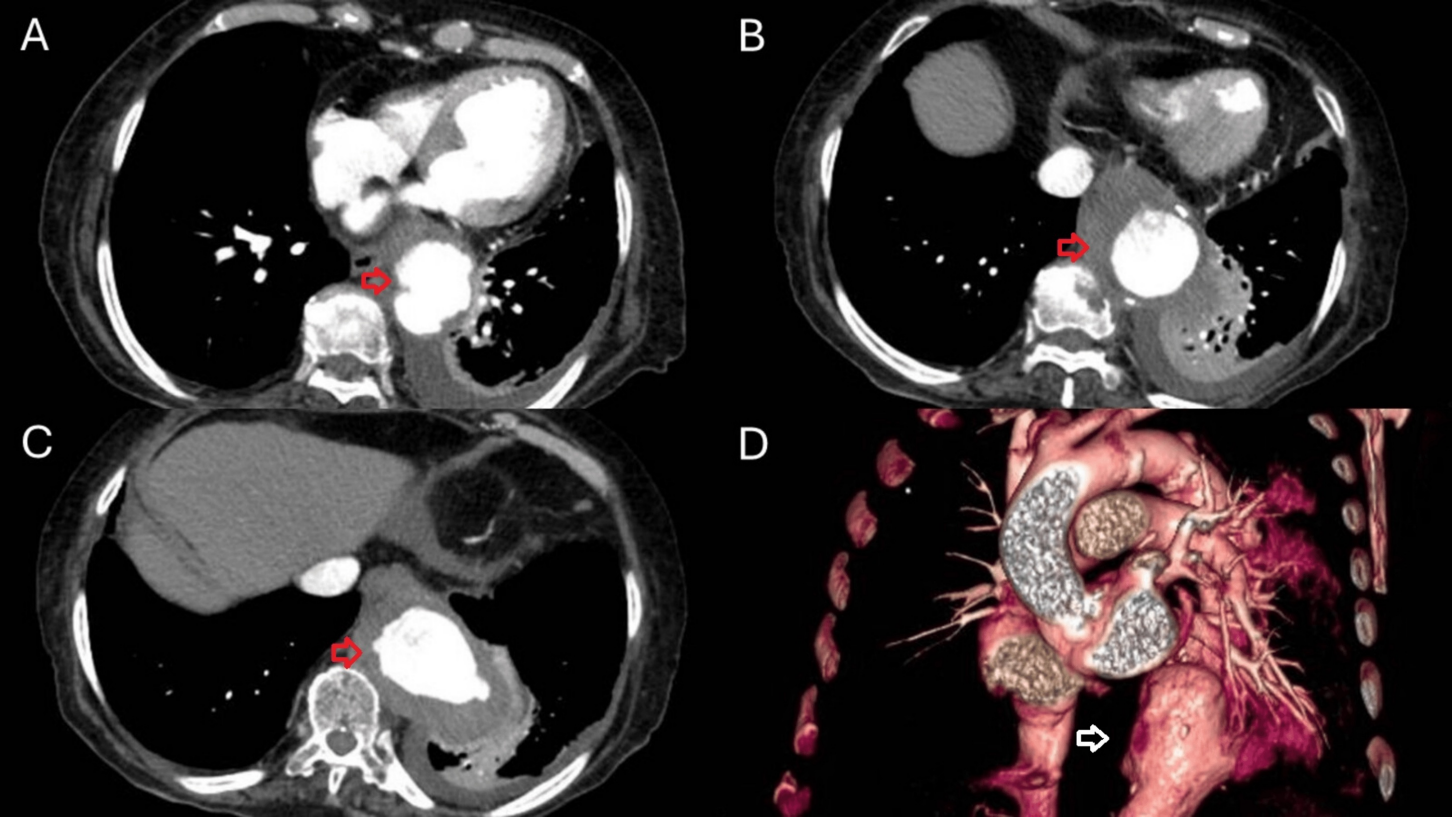
A-C: Contrast-enhanced CT showing aneurysm of the descending and diaphragmatic aorta with a rupture contained by a hematoma adjacent to the medial segment of the left lower pulmonary lobe (red arrows); D: Three-dimensional reconstruction showing aortic aneurysm of the descending aorta (white arrow)

Electrocardiogram only revealed sinus tachycardia. To complement the findings observed on chest radiography, we requested a contrast-enhanced thoracoabdominal computed tomography (CT) which revealed the following: aneurysm of the descending and diaphragmatic aorta, measuring 17 cm in length, with its greatest dilation being 9.3 x 5.7 cm at the level of the distal descending aorta. This was associated with multiple partially calcified ulcerated plaques and a rupture contained by a hematoma adjacent to the medial segment of the left lower pulmonary lobe with no evidence of frank contrast extravasation and a gas bubble in the descending aorta. Additionally, another focal aneurysm was identified at the level of the aortic isthmus, measuring 14 x 13 x 9 mm, and the thoracic aorta exhibited multiple partially calcified plaques and dilation of the aortic arch (Figure 2).

The cardiothoracic surgery and interventional radiology teams were consulted. After a thorough evaluation, the patient was deemed unfit for any interventional procedure due to the high risk of perioperative mortality, given her severe frailty and anatomical unsuitability for stent placement. Further consultations with cardiology and pulmonology specialists led to the decision to adopt a non-invasive conservative approach. Medications were initiated to manage heart rate and blood pressure, along with antitussives and laxatives to reduce episodes of increased intrathoracic and intra-abdominal pressure. Additionally, we administered 500 mg of tranexamic acid via nebulization to prevent further episodes of hemoptysis thrice daily. The geriatric team was also consulted and recommended continuing with the conservative approach, as well as initiating palliative care consultation and follow-up. The patient was monitored for five days under this medical management. We discussed her condition and prognosis with her and her family. After the period of observation, during which no further episodes occurred, she was discharged with plans for outpatient follow-up and palliative care.

## Discussion

The initial history and physical examination of hemoptysis should focus on identifying its most common causes and once excluded, other specific causes should be evaluated. Among rare causes, clinicians should be alert to life-threatening conditions such as complicated lung infections (e.g., lung abscess, necrotizing pneumonia), immunologic disorders (e.g., Goodpasture syndrome, granulomatosis with polyangiitis), pulmonary vascular diseases (e.g., pulmonary embolism), aorto-bronchial fistulas, among other conditions guided by the clinical scenario [2]. In the context of a thoracic aortic aneurysm, the clinical course is often silent unless complications occur. In some cases, symptoms like dyspnea, postprandial fullness, dysphagia, and chest pain can suggest that a non-ruptured aneurysm is enlarging. Most commonly, a ruptured aortic aneurysm will present with sudden, severe chest pain, dizziness, and hemodynamic instability. Very rarely, an aneurysm may present with hemoptysis, which could result from compression of the tracheobronchial tree or a broncho-aortic fistula in the context of a ruptured aneurysm, a life-threatening condition [3-5]. In this patient, the chest CT revealed a ruptured aortic aneurysm contained by a large hematoma, which made it difficult to identify an aorto-bronchial fistula. We believe this was the primary mechanism behind the patient's hemoptysis. Another less likely possibility could have been compression of the tracheobronchial tree due to the aneurysm.

After conducting a thorough literature review, we identified 23 reported cases of thoracic aneurysms presenting with hemoptysis [1-18]. Of these, 60% were male, with a median age of 71 years. Commonly reported comorbidities included type 2 diabetes, hypertension, chronic obstructive pulmonary disease, chronic smoking, and a history of chronic aortic aneurysms. While most aneurysms were found to have either an inconclusive etiology or were attributed to atherosclerosis, two were secondary to infections by *Aspergillus* and *Enterobacter*, and one was due to leukemic cell infiltration. In the current patient, we believe the etiology to be atherosclerosis, as the patient denied having a fever, the shock resolved without the use of antibiotics, and inflammatory markers were within normal range. In the literature, most cases of aneurysms are degenerative and associated with risk factors for atherosclerosis. Regarding interventions, eight patients underwent endovascular aortic repair with no perioperative deaths reported, while six patients underwent open repair, with two deaths reported post-surgery. The remaining cases did not undergo surgery due to refractory shock prior to intervention, poor overall condition, or being unfit for surgery; none of these patients survived.

A retrospective analysis of 157 octogenarians with aortic aneurysms found that 110 underwent surgery and 47 received palliative care, among patients undergoing surgical interventions, one-year mortality was 50% [19]. Reasons for palliative care were patient's own decision, profound shock state, and multiple comorbidities accompanied by a poor functional status. In another study evaluating outcomes in patients with non-ruptured abdominal aortic aneurysm undergoing palliative care, the median survival rate was 34 months with an estimated mortality of 40% [20]. Our patient presented with a ruptured thoracic aneurysm sheltered by a large hematoma which eventually ceased hemoptysis. Consultation for surgical or radiological intervention deemed the patient unfit for surgery. Contrary to the reported cases in which lack of surgical intervention resulted in almost 100% in-hospital mortality, our patient was discharged after five days with palliative management primarily aimed at reducing blood pressure and heart rate with subsequent follow-up from the palliative care team.

## Conclusions

In this case report, we described the diagnostic and therapeutic approach to a patient with hemoptysis in whom a ruptured aortic aneurysm with tracheobronchial communication was found. We highlight the importance of a careful clinical approach and accounting for the large spectrum of differential diagnoses for hemoptysis. Although in most cases it is non-life-threatening, accounting for any possibility could result in a timely diagnosis of rarer conditions that could threaten life such as a ruptured aortic aneurysm. 
